# The Evolution of Nerve-Sparing Radical Prostatectomy: Mechanisms of Injury, Economic Impact, and the Potential Value of Intraoperative Nerve Visualization

**DOI:** 10.3390/jcm15134981

**Published:** 2026-06-26

**Authors:** Michael Richards, Sahya Kabutogi, Sydney Lance, Thi Nguyen, Mark Bachir, Nathan McMahon, Connor W. Barth, David Yee

**Affiliations:** 1 College of Medicine, California Northstate University, 9700 W Taron Dr., Elk Grove, CA 95757, USA; sahya.kabutogi12304@cnsu.edu (S.K.); sydney.lance11491@cnsu.edu (S.L.); thi.nguyen11933@cnsu.edu (T.N.); mark.bachir6420@cnsu.edu (M.B.); david.yee@cnsu.edu (D.Y.); 2 Trace Biosciences, Beaverton, OR 97006, USA; nathan@trace-bio.com (N.M.); connor@trace-bio.com (C.W.B.)

**Keywords:** prostatectomy, iatrogenic, robotic-assisted surgery, pelvic plexus

## Abstract

**Background/Objectives:** Iatrogenic nerve injury is a significant challenge in urologic surgery, with radical prostatectomy posing a high risk due to complex pelvic neural anatomy. Despite advances in robotic-assisted and nerve-sparing techniques, postoperative urinary incontinence and erectile dysfunction remain prevalent, adversely affecting patients’ quality of life and imposing substantial healthcare costs. **Methods:** A narrative review was conducted using PubMed, MEDLINE, and the Cochrane Library (searches through February 2026) for studies on radical prostatectomy epidemiology, mechanisms of nerve injury, functional outcomes, and economic burden. Emerging intraoperative fluorescence imaging technologies, surgical strategies to mitigate iatrogenic nerve injuries, and the financial costs of post-prostatectomy complications were assessed. **Results:** Robotic-assisted radical prostatectomy now accounts for >80% of procedures in the United States, and has been associated in observational studies with improved early recovery of erectile function compared with open and laparoscopic approaches. However, the lack of real-time nerve visualization remains a limiting factor. Recent milestones (January 2026) include the Food and Drug Administration Investigational New Drug clearance for the nerve-specific fluorophore LGW16-03 (NerveTrace), which enables real-time identification of sub-millimeter nerve branches, and the 510(k) premarket clearance of Dendrite imaging (November 2025). **Conclusions:** Enhanced intraoperative nerve discrimination via molecularly targeted imaging has the potential to reduce iatrogenic complications and improve long-term functional and economic outcomes in prostate cancer surgery, although these benefits have yet to be demonstrated in prospective clinical and health-economic studies.

## 1. Introduction

Surgery is performed commonly in the United States, with approximately 40 million operations annually, and these surgeries account for 94% of all iatrogenic nerve injuries [1]. Iatrogenic nerve injury occurs across all surgical specialties, including general, thoracic, cardiac, urologic, plastic, colorectal, spinal, neuro-, vascular, and otolaryngologic surgery [1]. Unlike other critical tissues, such as blood vessels, nerve injuries cannot be repaired with reliable functional improvement [2]. The peripheral nervous system plays a critical role in connecting the central nervous system with the end organs required for voluntary and involuntary bodily functions and in maintaining biochemical homeostasis [3]. Therefore, iatrogenic nerve injury is often permanent and, in the setting of peripheral nerve injury, leads to disabled motor and protective sensory function that can limit patients’ ability to work, participate in activities of daily living, and predispose them to injury. Thus, nerve injury prevention strategies are the only known method of successfully counteracting iatrogenic peripheral nerve injury [2].

Surgeons primarily rely on their anatomical knowledge, visual white-light guidance, preoperative imaging guidance technologies, and, occasionally, low-amperage nerve stimulators (i.e., electromyographic monitoring) to identify important peripheral nerves [4]. In the setting of minimally invasive surgeries (e.g., laparoscopic, robotic), peripheral nerves are at risk because of limited visualization and assumptions about anatomical landmarks. Factors such as trauma, tumors, prior surgery, congenital anomalies, and radiation therapy create inconsistency in the location of anatomical landmarks, creating a challenge for surgeons in identifying critical nerve structures for preservation. However, to date, there are limited Food and Drug Administration (FDA)-approved options to enhance a surgeon’s real-time visual recognition of nerve tissue during a procedure.

Several novel fluorescence imaging solutions are being developed to address this unmet clinical need, which provide real-time intraoperative visualization of nerves with high specificity and sensitivity. The Dendrite Imaging, Inc. imaging camera uses near-ultraviolet (NUVL) excitation to induce autofluorescence of neural structure, and was granted 510(k) premarket clearance by the FDA in November 2025 [5]. To date, this technology has been evaluated in the setting of thyroidectomy, inguinal hernia repair, and other surgeries. Each study showed that autofluorescence improved visualization of relevant neural structures, and none of the patients experienced symptoms typically associated with iatrogenic nerve injury [6,7,8]. However, the technology has only been evaluated in small samples or retrospective studies, and not been tested against a control, making it difficult to evaluate its relative impact on patient outcomes. Trace Bioscience’s product NerveTrace is a novel small molecule nerve-labeling fluorophore. After receiving Investigational New Drug (IND) clearance from the FDA in January 2026, Trace is preparing to initiate its first-in-human clinical studies in June 2026 [9].

Radical prostatectomies (RPs) pose a significant risk of iatrogenic nerve injury due to the dense, intricate anatomy of autonomic and somatic nerves coursing through the pelvis [10]. These surgeries require dissection in regions where critical neural structures are both difficult to visualize and highly susceptible to traction, thermal, or transection injuries [11]. The resulting urinary incontinence and sexual dysfunction can be profound and contribute to long-term reductions in quality of life [12]. Beyond the clinical impact on patients, these injuries contribute to prolonged recovery times, increased healthcare utilization, and substantial economic burden for hospitals and payers. Given the high incidence of nerve injuries after prostatectomy as well as the rising cases of prostate cancer in the aging population, the purpose of this narrative review is to examine the prevalence of radical prostatectomies in the United States and worldwide, the risk of iatrogenic nerve injury associated with these surgeries, the current surgical techniques applied to mitigate complications, and the costs associated with the surgeries and the associated nerve injuries. This information will be used to inform a use case for current and future solutions, including novel fluorescence imaging technologies.

## 2. Materials and Methods

This narrative review is based on a targeted, non-systematic literature search that was conducted to identify relevant studies examining iatrogenic nerve injury in the context of radical prostatectomy. The electronic databases PubMed, MEDLINE (via PubMed), and the Cochrane Library were searched from September 2025 to February 2026 using the following keywords, both individually and in combination: “prostatectomy”, “robotic”, “minimally invasive”, “laparoscopic”, “nerve injury”, “iatrogenic”, and “cost”. Search results were screened by title and abstract to identify articles focusing on nerve injury associated with this surgical procedure. Eligible studies included original research articles, systematic reviews, and meta-analyses published in English. Case reports, conference abstracts, and non-peer-reviewed sources were excluded. References from included articles were also manually reviewed to capture additional relevant studies. These data were used to compile a narrative review of radical prostatectomy-related nerve injury, and its associated outcomes and costs. The selection process was not designed or reported as a formal systematic review, and no standardized risk-of-bias assessment was performed; instead, we synthesized the literature qualitatively, emphasizing higher-quality observational studies, randomized trials, and meta-analyses where available.

## 3. Epidemiology and Trends in RP

Prostate cancer (PCa) is the most common non-cutaneous cancer affecting men, and the second leading cancer cause of mortality behind lung cancer, with 313,780 new cases and 35,770 deaths in 2025 per the Surveillance, Epidemiology, and End Results (SEER) database [13]. The majority of diagnosed PCa patients present with localized disease [13]. Numerous treatment modalities are available, including active surveillance, focal therapy, radiation, and surgical resection. Patients and their urologists should select the optimal treatment based on risk stratification, defined by tumor size, pathologic grade group, and PSA level [14]. Per National Comprehensive Cancer Network (NCCN) guidelines, RP can be used as initial therapy for PCa of any risk group in patients with greater than 10-year life expectancy, with observation or radiation preferred in patients who do not meet these criteria [15]. RP is a standard surgical treatment option for clinically localized disease, and significantly decreases PCa-specific mortality and metastatic progression [14]. The National Surgical Quality Improvement Program (NSQIP) of the American College of Surgeons (ACS) reports 43,025 RP patients between 2008 and 2015 [16]. This number includes all RP approaches, including open, laparoscopic, and robotic. A more recent study by Freitas et al. analyzed the National Cancer Database, and found a total of 610.762 patients who underwent RP in the US between 2006–2020, with 211,799 occurring between 2016–2020 [17]. Another study analyzing National Inpatient Sample (NIS) and Healthcare Utilization Project (HCUP) data from 2010–2015 reports 69,414 patients in the United States who underwent RP, with 405 of these being laparoscopic RP (LRP), 52,151 robot-assisted RP (RARP), and 16,858 open RP (ORP) [18].

Notably, there has been a significant increase in the use of RARP since 2010, as Mukherjee and Kamal found the proportion of procedures done robotically increased from 66.69% in 2010 to 83.27% in 2015 [18]. Interestingly, when data were adjusted for covariates, RARP demonstrated significantly lower rates of complications (Odds Ratio: 0.73, 95% Confidence Interval: 0.68–0.78) as well as reduced length of hospital stay (mean for RARP: 1.90 vs. ORP: 2.75 days, *p* < 0.0001) [13]. RARP is becoming more commonly elected as improvements in prostatectomy-associated outcomes like continence and potency recovery are discerned [18]. Ploussard et al. found that the proportion of RARPs increased from 39.8% (*n* = 7621) in 2015 to 52.6% (*n* = 10,577) in 2019, and the proportion of ORPs decreased from 34.4% (*n* = 6592) in 2015 to 24.5% (*n* = 4931) in 2019 [19]. A 2021 study, using administrative claims data from the Optum Labs Data Warehouse in the United States (US) and Hospital Episodes Statistics in England, examined 66,879 RP cases in England and 79,358 in the US from 2005 to 2017 [20]. This study found a significant increase in the adoption of robotic techniques in both countries, although England’s adoption was lower than that of the US—43% of hospitals in England and 58% in the US performed robotic surgery by 2017 [20]. While there’s a varying number of reported cases, overall, the presented data indicate that RP is a very commonly performed procedure, and the adoption of robotic techniques has been steadily growing both in the US and in Europe.

Importantly, studies investigating oncologic outcomes across surgical approaches indicate similar or improved recurrence and positive surgical margin rates in RARP. A study by Labban et al. showed that biochemical recurrence rates within a 10-year window for RARP, LRP, and ORP were 28%, 37% and 32%, respectively [21]. Kim et al. determined that RARP had significantly lower rates in comparison to LRP and ORP, (RR 0.672, 95% CI 0.505–0.895; RR 0.713, 95% CI 0.587–0.869), while there was no significant difference between LRP and ORP (RR 1.060, 95% CI 0.771–1.452) [22]. However, an older study from 2015 showed that, when adjusted for preoperative variables, 5-year biochemical recurrence-free survival rates were similar in retropubic and RARP patients (48.5% vs. 56.9%, *p* = 0.131) [23]. The difference between these data could suggest improvement in robotic techniques or be a function of differences in study design. Overall, these data indicate that robotic approaches are at least comparable to open and laparoscopic ones in terms of oncological control.

## 4. Mechanisms of Iatrogenic Nerve Injury in RP

The standard RP approach for localized PCa involves the removal of the prostate gland, seminal vesicles, part of the vas deferens, and often the bladder neck [11,23]. Following excision, the urinary tract is reconstructed by lowering the bladder into the pelvic floor and anastomosing the bladder to the urethra [23]. Despite substantial advancements in prostatectomy techniques and their contributions to enhanced survival, RP disturbs the pelvic floor integrity, damages the autonomic and sensory nerve structures in the region, and is associated with complications such as urinary incontinence (UI) and erectile dysfunction (ED), both of which significantly compromise quality of life [24].

### 4.1. Relevant Neuroanatomy

Erections are produced when blood fills the sinusoids in the ischiocavernosus and bulbospongiosus muscles in response to sexual stimulation and arousal [25]. This inflow then causes restriction of venous outflow from the corpora cavernosa, which maintains the erection or tumescence [25]. This process is mediated by parasympathetic activity, which causes penile arterial dilation and smooth muscle relaxation in the corpora cavernosa [25]. The arteries involved are three branches of the internal pudendal artery: the corpora cavernosa are supplied by the dorsal and deep arteries of the penis, and the corpora spongiosum is supplied by the dorsal artery of the penis and the arteries of the bulb of the penis [25]. The innervation is supplied by nerves originating from the sacral S2–4 nerve roots [25]. The prevertebral plexus enters the pelvic region to form the hypogastric nerves, which are located medial to the internal iliac artery and pass through the pelvic inlet [25]. The hypogastric nerves meet the pelvic splanchnic nerves to form the pelvic or inferior hypogastric plexus [25]. These nerves branch further into the prostatic plexus and the cavernous nerves, which compose the neural portion of the neurovascular bundles (NVBs). Importantly, in RP, the cavernous nerves lie posterolateral to the prostate, between the outer levator fascia and the inner prostatic fascia, and are at significant risk of injury during surgery [11]. There can be slight variations in the location of these NVBs, and they are not readily visible under white light illumination or magnification, which can make precise dissection more challenging [26]. All of these nerves carry parasympathetic fibers that mediate the vasodilation involved in producing and maintaining erections [25]. Disruption of these nerve fibers through mechanical or thermal injury can result in impaired signaling and ED.

The lower urinary tract (LUT) is innervated by three sets of peripheral nerves [27]. The pelvic nerves arise in the sacral spinal cord and provide parasympathetic stimulation to the detrusor wall layers and urethral smooth muscle, resulting in bladder contraction and relaxation of the urethra [27]. The lumbar sympathetic nerves inhibit bladder contraction and excite the bladder base and urethra, allowing for the storage of urine [27]. These nerves pass through the sympathetic chain ganglia to the inferior mesenteric ganglia, the hypogastric nerves, and finally to the pelvic ganglia before synapsing at their target structures [27]. Finally, somatic control of the external urethral sphincter and pelvic floor muscles is controlled by the pudendal nerves [27]. These are paired nerves that emerge from the S2–4 spinal cord and exit the pelvis through the greater sciatic foramen before coursing into the perineum [27,28]. Damage to any of these nerves can result in various forms of UI and decreased patient quality of life [28].

### 4.2. Proposed Mechanisms of Urinary Incontinence from RP

Urinary incontinence is defined by the International Continence Society (ICS) as a complaint of any involuntary urine outflow [29,30]. The mechanism by which UI occurs after RP is complex; however, it is thought to be secondary to damage to the intraprostatic nerves and the external urethral sphincter and their innervation [31]. In addition, because many RP approaches involve removal of the bladder neck, including the internal urethral sphincter smooth muscle, the nearby striated external urethral sphincter and its innervation can be injured [23,32]. If the external sphincter or its innervation is injured, patients may develop stress urinary incontinence [23]. Although mechanical damage to the external sphincter is often considered the primary driver of post-RP UI, studies have shown that electrical nerve stimulation provides additional benefit in continence-recovery regimens [31]. This suggests that neural inputs play an important role in continence recovery post-RP, and that further neuroprotective measures are warranted.

### 4.3. Proposed Mechanisms of Erectile Dysfunction from RP

**Figure 1:**
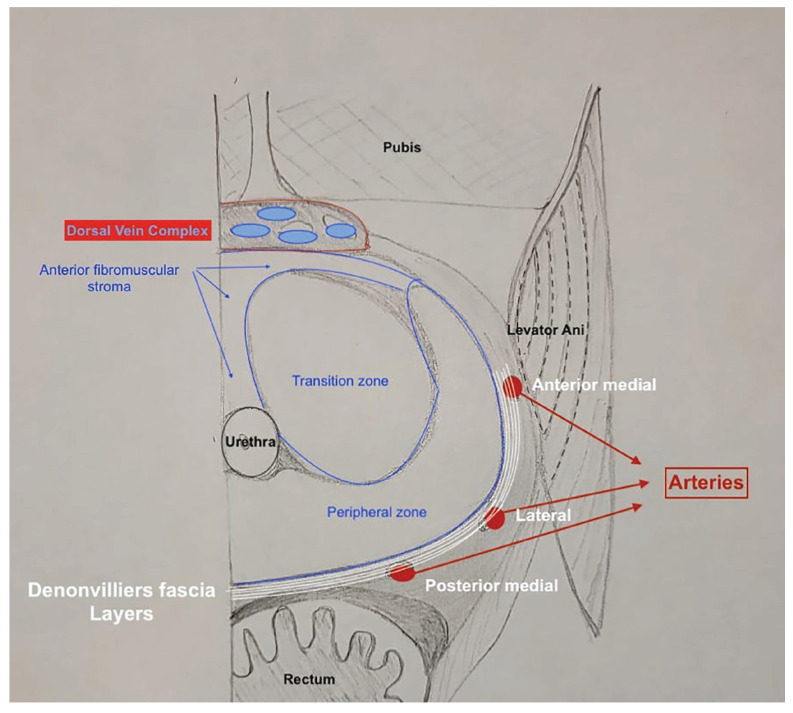
Prostate anatomy describing the arterial landmarks used to guide the nerve-sparing on the right side. Adapted from Moschovas and Patel (2021) [32]. Reproduced with permission from Dr. Marcio Moschovas, International Journal of the Brazilian Society of Urology; published by Sociedade Brasileira de Urologia, 2021.

ED occurs via damage to the NVBs containing the cavernosal nerves or their blood supply during prostate dissection from the Denonvilliers fascia, which separates the prostate from the rectum [32]—Figure 1. This is occasionally performed intentionally or unilaterally when necessary to achieve optimal oncologic control [11]. Anatomical studies demonstrate that the cavernous nerves form a meshwork around the prostate, and immunostaining of nerve fibers indicates that approximately 25% of the nerves are located in the anterior region [24,26]. During RP, mechanical or thermal injury of the NVB typically occurs during lateral or apical dissection [32]. As these nerves provide the parasympathetic signaling that drives vasodilation of the internal pudendal artery and its branches, damage to them reduces penile hyperemia in the corpus cavernosum, impairing the ability to achieve erections [24].

In certain cases, pelvic lymph node dissection (PLND) is utilized to remove potential sentinel lymph nodes for control of PCa metastasis. The clinical utility of PLND is debated in some circles due to increased surgical time and unclear oncologic benefit; however, it can be considered in higher-risk patients [33,34,35]. The standard template includes dissection of the external and internal iliac and obturator nodes [35]. While uncommon, sexual dysfunction such as ED can occur due to damage to the hypogastric nerves during PLND and the neural hammock due to their proximity to the internal iliac vessels and the prostate [25,34,35].

## 5. Functional Outcomes

To accurately capture the prevalence of nerve injury during RP, literature assessment of the associated comorbidities provides the most complete estimate. The reported prevalence of both UI and ED after RP varies widely across the literature. A cross-sectional study from Brazil found that post-radical prostatectomy incontinence (PRPI) was observed in 46.7–80.3% of 152 men, depending on the assessment method (pad test, pad use, and item three of the International Consultation on Incontinence Questionnaire—Short Form (ICIQ-SF)) [36]. A similar study was conducted in Norway, reporting a prevalence of PRPI of 74% 1 year postoperatively, 40% requiring daily pads, and 34% experiencing occasional leakage without pads [37]. For ED, meta-analyses have found ranges as wide as 14–90% [38], but high-quality studies such as the Prostate Cancer Outcomes Study (PCOS) estimate that 78–87% of men who undergo RP experience some level of ED [39].

### 5.1. Robotic-Assisted vs. Traditional Open or Laparoscopic RPs

RARP, with its high-resolution endoscopes and articulated forceps, allows for detail-oriented maneuvers and dexterity within the narrow area in the pelvis, and many studies have demonstrated improved preservation of erectile function compared to that of open or laparoscopic RP [38,39]. A systematic review by Moretti et al. found that robotic approaches were associated with higher rates of erectile function at 1, 3, 6, 12, and 18 months compared with open and laparoscopic approaches [40]—Table 1. Urinary continence may be less variable across methods, as noted by Haglind et al., who found no significant difference in incontinence rates between open and robotic approaches [41]. Interestingly, a meta-analysis reports that urethral length preservation was associated with improved post-op continence. In this study by Xiong et al., 61.9% of patients who underwent maximal urethral length preservation were continent at 1 month post-op, and 92% at 12 months, compared to 39.4% and 86.7% in those who did not (Z = 3.62, *p* = 0.003, OR = 3.10, 95% CI:1.68–5.73; Z = 2.34, *p* = 0.019, OR = 2.10, 95% CI:1.13–3.90) [42].

### 5.2. Nerve-Sparing Techniques

Nerve-sparing (NS) techniques have been developed in an effort to improve functional outcomes after RP. Multiple techniques exist and are primarily classified by whether the dissection is intrafascial, where dissection takes place internal to the prostatic fascia, anterior to Denonvilliers’ fascia, or interfascial, where the plane is developed between the leaves of the prostatic fascia and medial to the NVB [10,11]. Regardless of the specific technique, NS is achieved through cold or athermal dissection and control of the prostatic pedicle, reducing traction on the NVB [10,11]. While initially designed to preserve erectile function after RP, NS has also been observed to improve urinary continence rates. A large retrospective cohort study of 431 patients who underwent RARP found that patients in the NS cohort had lower mean urine leakage volume at 1 and 2 months (16.40 g vs. 49.44 g, *p* < 0.001; 13.60 g vs. 35.45 g, *p* = 0.009), however no significant differences were seen at 6 or 12 months [43]. It remains a matter of debate whether these benefits derive from nerve preservation itself or from intact peri-prostatic supporting structures. Evidence is emerging that urinary continence is improved with intact supporting tissues around the prostate, since these tissues confer a higher maximum urethral closure pressure after NS surgery [12].

In the retrospective cohort of 431 RARP patients in Hong Kong, patients who underwent NS or non-NS procedures were examined regarding their postoperative outcomes, including ED and UI. ED scores were judged based on the International Index of Erectile Function-5 (IIEF-5), and the study demonstrated that patients with bilateral NS had a higher mean postoperative IIEF-5 score after 2 and 3 months compared to non-NS patients. Interestingly, patients with bilateral NS compared to unilateral NS had better postoperative function and outcomes [43].

### 5.3. Retzius-Sparing Procedure

The Retzius-sparing procedure was developed to remove the prostate from the posterior aspect of the bladder, leaving the anterior bladder and supporting structures intact. This technique preserves the high bladder and urethral position, resulting in improved continence outcomes, particularly in the early course of recovery [12]. A study comparing continence recovery rates between patients who underwent conventional versus Retzius-sparing RP found 9% and 45% continence, respectively, at 1 month post-RP, with these rates improving to 77% and 98%, respectively, at 6 months post-op [44]. Critical anatomy-sparing techniques have been proven to improve both recovery rate and speed for functional outcomes post-RP. Thus, new technologies designed to improve tissue sparing can anticipate similar improvements and warrant further development.

### 5.4. Therapeutic Implications and Postoperative Rehabilitation After RP

Prior to undergoing RP, surgeons should clearly outline the potential complications of surgery and the treatments, including ED, available to address them. Penile rehabilitation is the strategic application of interventions that address erectile function following RP or radiation therapy for PCa [45]. While data is mixed, it is generally perceived that this process should be initiated either prior to surgery or as soon as possible post-op [45]. Pre- and post-op erectile function should be established using validated questionnaires, such as the IIEF-5 score, to establish a baseline, and monitored over time to assess improvement [45].

First-line therapies include lifestyle changes, pelvic floor muscle exercises, and PDE-5 inhibitors [45,46]. Lifestyle changes should be focused on addressing risk factors for vasculogenic ED, such as diabetes, hypercholesterolemia, hypertension, and cigarette smoking [47]. Smoking cessation, and improved control of blood glucose, blood pressure, and cholesterol can improve erectile dysfunction and reduce the impact of non-surgery-related ED [48]. It is important to note that 35% of patients with vasculogenic ED do not respond to PDE-5 inhibitors, and some patients struggle with compliance due to side-effects [49]. In these patients, it is reasonable to pursue second-line treatments such as low-intensity shockwave therapy, intracavernosal injections, vacuum erection devices, and intraurethral or topical application of prostaglandin E1 analogues, such as Alprostadil [48]. For patients who do not respond to these second-line therapies, penile prosthesis surgery can be offered. Of note, diabetics can be as much as twice as likely to fail second-line therapies and undergo surgery [45,46].

### 5.5. Emerging Regenerative Approaches for Vasculogenic ED

Several autologous cell therapies are currently under investigation in the setting of both vasculogenic and RP-induced ED; however, they have only been studied in a limited capacity, and no standardized protocols currently exist [49]. Platelet-rich plasma (PRP) injections are believed to assist in the regeneration and restoration of cavernous nerve function following RP, as well as possibly inhibiting fibrosis in the corpus cavernosa [50,51,52]. Various studies have shown promising results, though further research is needed. Ding et al. and Wu et al. studied the effects of PRP on cavernous nerve function after damage in a rat model and found that intracavernosal pressure was significantly higher in rats that received PRP injections at the injury site. Additionally, there was increased preservation of cavernous nerve myelination, which theoretically indicates improved erectile function [50,51]. A clinical trial by Epifanova et al. found that IIEF-5 scores improved following 3 weekly intracavernosal injections, and that the injections were well-tolerated. However, there was no control group for comparison, so no definitive efficacy claims can be made [52].

Heterologous and autologous stem cell therapy have also been investigated. The mechanism by which these therapies act is not well understood, but it is believed that paracrine release of bioactive factors to endothelial tissue due to their proangiogenic, anti-inflammatory, anti-apoptotic, and anti-fibrotic properties [53,54,55]. Multiple studies, including several clinical trials that established tolerability and safety have been carried out to date [56,57,58,59,60]. These trials have shown improvement in IIEF-5 scores, peak systolic velocity to suggest improved blood flow [56,57,58,59,60]. However, a study by Haahr et al. found that patients with concomitant UI did not exhibit any improvement in erectile function, though the sample was too small to be generalizable [59].

Similarly to stem cell therapy, peripheral blood mononuclear cell therapy (PBMNC) is believed to act via paracrine mechanisms to improve erectile function [49,61]. These treatments differ slightly from stem cells in their secretome’s ability to induce cellular proliferation. Notably, apoptotic PBMNCs have been shown to be angiogenic, induce vasodilation, enhance re-epithelialization and modulate the immune system by paracrine mechanisms [49,61,62]. A clinical trial by Yiou et al. showed a dose-sensitive improvement in erectile function, peak systolic velocity, and penile nitric oxide release following intracavernosal injection. However, erectile function at follow-up scores at a mean of 62.1 ± 11.7 months was lower than at 1 year, suggesting the need for multiple injections [63]. These investigational therapies, while promising, do have their limitations. For example, diabetic patients may not be ideal candidates due to a deficiency in vascular regenerative cells and angiogenic capacity, with some authors suggesting that structural dysfunction of mesenchymal stem cells from diabetic pts may limit their potential therapeutic use [64,65]. Further research is warranted to fully elucidate the potential impact of these treatments and possibly develop structured protocols for their use.

## 6. Economic Burden of RP-Related Nerve Injury

Given the diverse landscape of surgical techniques for RP and the high risk of nerve damage, understanding the economic impacts of each option and the potential associated nerve injury is critical to informed care. In comparison to laparoscopic and open techniques, RARP is significantly more expensive, due to the cost of robot acquisition and maintenance, and the additional disposables used in surgery. ORPs were reported to be the least expensive and most cost-effective form of RP, costing hospitals an average of $487 and $1726 lower than laparoscopic and robotic techniques [66]. While there are initial cost disadvantages to RARPs, there is evidence to suggest lower long-term costs to patients due to the perioperative advantages and reduced postoperative complications [67]. A study on the healthcare costs of 13,360 RP patients showed higher initial hospitalization costs for RARP than ORP (mean difference, $2367; *p* < 0.001), but lower total 1-year cumulative costs (mean difference −$383; *p* = 0.60) due to lower rates of emergency department and hospital outpatient visits [67]. This reduction in healthcare service use among RARP patients resulted in a mean additional savings of $2929 (*p* < 0.01) compared with ORP patients [67]. A similar study from the UK found that RARP was more cost-effective and yielded higher quality-adjusted life years (QALYs) than LRPs, saving approximately $2350 on average and yielding an additional 0.24 QALYs [21]. Compared to ORPs, however, RARPs cost more (+$693) but still yielded additional +0.12 QALYs and an incremental cost-effectiveness ratio (ICER) of $5653/QALY that was well below the authors’ “willingness to pay” threshold of $39,503/QALY [37]. These trends were similar to those observed in Japan, with RARP associated with significantly fewer complications, shorter postoperative length of stay, lower postoperative costs, and lower costs excluding the operation, as compared with 7202 ORP, 2483 LRP, and 2126 RARP cases [68]. However, this study also noted that concerns regarding the profitability of RARP warrant further investigation, as hospitals would need to perform at least 100 RARPs annually to offset the high cost of robotic console acquisition and maintenance [68]. The reduction in long-term costs of RARP suggests that increased surgical precision can be associated with a more cost-effective procedure for both patients and hospital systems [21,67,68]. However, these data pertain specifically to robotic vs. open technique and do not provide direct evidence that nerve-specific fluorescence imaging will be cost-effective. Whether tools that enhance intraoperative nerve visibility can achieve similar or additional cost offsets remains a hypothesis that requires formal health-economic modeling and prospective evaluation.

## 7. Discussion

Treatment options for PCa range from active surveillance in low-grade disease or older patients to more definitive options like RP in individuals with localized disease and longer life expectancy [15]. Physicians may endorse surgical interventions for younger patients because aggressive operative intervention becomes increasingly contraindicated with advanced age [15]. Common complications of RP include ED and UI, which can be debilitating [16,36,38,39,40]. For RP, the surgical objective must extend beyond oncological control, and surgeons are challenged with achieving functional outcomes to prevent devastating, lifelong reductions in quality of life.

Given the unique, complex network of parasympathetic, sympathetic, and somatic nerves embedded within the periprostatic fascia, the development of NS techniques serves to optimize functional outcomes after RP [12,30,31,32,35,37]. RPs are inherently at high risk of nerve injuries since NVB fibers are microscopic and often indistinguishable under the naked eye or standard surgical magnification [10]. Current literature appears to suggest decreased complication rates, such as ED and UI, with the application of NS techniques, such as urethral length preservation or Retzius-sparing procedures [12,30,31,32,35,37,44]. These improved outcomes are achieved through the delicate handling of NVBs and critical proximal architecture of the bladder or reproductive organs.

With the ongoing refinements of NS techniques and the widespread adoption of laparoscopic and robotic approaches, rates of oncologic clearance and biochemical recurrence are least comparable in RARP versus other techniques [21,22,23]. However, postoperative ED and UI continue to exist at high rates, which implies that surgical evolution may have reached a plateau in terms of mechanical dexterity and magnification [16,30,31,32]. However, tissue differentiation remains a task for surgeons to carefully identify microscopic neural fibers.

Hence, our collective evidence evaluating the functional and financial burden of managing RP complications showcases the unmet clinical need for enhanced intraoperative visualization. Fluorescence-guided technologies, such as Dendrite and NerveTrace, may help address this gap by providing real-time identification of peripheral nerves [4,5,6,7,8,9]. Early preclinical and retrospective data suggest that nerve-targeted fluorophores can generate high-contrast visualization of small-caliber peripheral nerves, but clinical outcome data for RP are not yet available. Enabling surgeons to identify and preserve autonomic and somatic nerve fibers that are otherwise indistinguishable under white light could theoretically enhance the precision of NS approaches and reduce iatrogenic injury rates, but these potential benefits must be demonstrated in future prospective trials. Given that the current techniques can result in significant postoperative dysfunction, integrating nerve identification tools like NerveTrace and Dendrite represents a possible next step towards optimizing both surgical outcomes and patient quality of life.

These technologies could be incorporated into a broader therapeutic approach that would include established treatments like PDE-5 inhibitors, penile rehabilitation, and others, as well as emerging treatments in PRP-, SCT-, and PBMNC-based ones [46,47,48,49,50,51,52,53,54,55,56,57,58,59,60,61,62,63,64,65]. As further studies are carried out, and more treatments are incorporated into guidelines, physicians and patients would have more options available to them. This could create an environment where surgical recovery can be more multimodal, customizable, and patient-centered. While these are potentially impactful innovations, barriers such as cost may persist.

Our review, while comprehensive, has several limitations. Financial constraints prevented all prevalence and cost data from being obtained from a single verified database, and some economic information was derived from secondary sources. Much of the data from national databases could not be accessed due to the authors’ institution not being associated with the NSQIP or HCUP, thereby preventing us from obtaining the most up to date data. This, in turn, may introduce variability and reduce external validity. Additionally, the authors do not possess advanced experience in health economics, which limits the depth of cost-analysis discussions, and finally, this study focuses on RP alone, restricting the generalizability to other surgical domains in which these imaging technologies may have clinical application.

While we anticipate increased initial procedural costs with the incorporation of advanced intraoperative nerve visualization tools, any potential for this expense to be offset by reductions in the cumulative costs of managing chronic UI and ED remains speculative at present, and should be formally evaluated in future health-economic analyses.

Future studies should expand this scope of analysis to include a broader array of high-risk surgeries and perform detailed economic modeling of complication reduction to ultimately validate the clinical effectiveness of intraoperative nerve visualization technologies after prospective multi-institutional trials. Demonstrating a measurable reduction in postoperative neurologic deficits and improvement in quality-of-life outcomes will be critical in establishing both the clinical and economic value of NerveTrace and similar innovations in modern surgical practice.

## 8. Conclusions

Ongoing refinements of surgical techniques continue to focus on balancing oncologic clearance with functional erectile and urinary function by promoting nerve preservation. While the success of robotic and NS techniques in reducing post-operative ED and UI suggests they are a worthwhile pursuit, the continued prevalence of these complications indicates that further improvement is necessary. Economically, robotic and NS techniques have the potential to improve cost-effectiveness based on the reduced rates of complications that require chronic postoperative treatment. By improving the precision with which surgeons operate, nerve-visualization technologies such as Dendrite and NerveTrace may ultimately yield additional gains in functional and economic outcomes. However, this possibility remains hypothetical and will require confirmation in prospective trials accompanied by robust health-economic evaluation.

## Figures and Tables

**Table 1:** Mean overall erectile function rates across RP approaches. Data summarized from a reverse systematic review by Moretti et al. including 131,350 patients undergoing open, laparoscopic, and robotic radical prostatectomy; erectile-function recovery was most commonly defined as erections sufficient for intercourse using validated questionnaires, although definitions varied across included studies.

	1 Month	3 Months	6 Months	12 Months	18 Months+
ORP	16%	22%	30%	41%	58%
LRP	12%	26%	44%	55%	52%
RARP	35%	42%	54%	59%	67%

Values represent pooled mean rates across heterogeneous cohorts and should be interpreted cautiously given differences in baseline characteristics, surgical techniques, follow-up duration, and criteria for erectile-function recovery.

## Data Availability

No new data were created or analyzed in this study. Data sharing is not applicable to this article.

## Abbreviations

The following abbreviations are used in this manuscript:

FDA	Food and Drug Administration
RP	Radical Prostatectomy
PCa	Prostate Cancer
SEER	Surveillance, Epidemiology, and End Results
NCCN	National Comprehensive Cancer Network
ACS	American College of Surgeons
NIS	National Inpatient Sample
HCUP	Healthcare Utilization Project
LRP	Laparoscopic Radical Prostatectomy
RARP	Robot-Assisted Radical Prostatectomy
ORP	Open Radical Prostatectomy
UI	Urinary Incontinence
ED	Erectile Dysfunction
NVBs	Neurovascular Bundles
LUT	Lower Urinary Tract
ICS	International Continence Society
PLND	Pelvic Lymph Node Dissection
PRPI	Post-Radical Prostatectomy Incontinence
ICIQ-SF	International Consultation on Incontinence Questionnaire-Short Form
PCOS	Prostate Cancer Outcomes Study
NS	Nerve Sparing
IIEF-5	International Index of Erectile Function-5
QALYs	Quality-Adjusted Life Years
ICER	Incremental Cost-Effectiveness Ratio
PRP	Platelet-rich plasma
SCT	Stem cell therapy
PBMNC	Peripheral blood mononuclear cell

## References

[1] Burke S., Shorten G.D. (2009). When pain after surgery does not go away. Biochem. Soc. Trans..

[2] Li R., Hettinger P.C., Machol J.A., Liu X., Stephenson J.B., Pawela C.P., Yan J.G., Matloub H.S., Hyde J.S. (2013). Cortical plasticity induced by different degrees of peripheral nerve injuries: A rat functional magnetic resonance imaging study under 9.4 Tesla. J. Brachial Plex. Peripher. Nerve Inj..

[3] Olsson Y. (1990). Microenvironment of the peripheral nervous system under normal and pathological conditions. Crit. Rev. Neurobiol..

[4] Echeverri A., Flexon P.B. (1998). Electrophysiologic nerve stimulation for identifying the recurrent laryngeal nerve in thyroid surgery: Review of 70 consecutive thyroid surgeries. Am. Surg..

[5] Dendrite Imaging (2025). Dendrite Imaging Announces FDA Clearance of Nerve-Imaging Surgical Camera. https://dendriteimaging.com/news?view=article&id=49%3Adendrite-imaging-announces-fda-clearance-of-nerve-imaging-surgical-camera&catid=10.

[6] Harraca J., Aleman R., Schor M., Melgar J.I., Marozzi F., Eichman S. (2025). Introducing autofluorescence technology for inguinal nerve visualization in open anterior hernia surgery: A step forward in surgical precision and safety. Hernia.

[7] Dip F., Aleman R., Dip H.R., Rancati A., Eiben G., Marinelli F., Ghiselli J., Sinagra D., Aleman J., Rosenthal R.J. (2025). Enhancing Recurrent Laryngeal Nerve Detection with Neural Autofluorescence in Thyroid Surgery. J. Am. Coll. Surg..

[8] Dip F., Aleman R., Marinelli F., Guiselli J., Rosenthal R., Rancati A., Sinagra D. (2025). High-precision identification of sensory and motor branches of the recurrent laryngeal nerve via autofluorescence system in thyroid surgery. Cureus.

[9] Henderson E.R. Study of LGW16-03 to identify nerves. ClinicalTrials.gov. Identifier: NCT07385430. NCT07385430.

[10] Li X., Wu J., Cai Q., Pan J., Meng Q., Zhang P., Xu Y., Zhai L. (2021). The distribution pattern of periprostatic neurovascular bundles examined with successive celloidin slices. BMC Urol..

[11] Walz J., Epstein J.I., Ganzer R., Graefen M., Guazzoni G., Kaouk J., Menon M., Mottrie A., Myers R.P., Patel V. (2016). A critical analysis of the current knowledge of surgical anatomy of the prostate related to optimisation of cancer control and preservation of continence and erection in candidates for radical prostatectomy: An update. Eur. Urol..

[12] Kumar A., Patel V.R., Panaiyadiyan S., Seetharam Bhat K.R., Moschovas M.C., Nayak B. (2021). Nerve-sparing robot-assisted radical prostatectomy: Current perspectives. Asian J. Urol..

[13] National Cancer Institute Cancer of Any Site. Surveillance, Epidemiology, and End Results Program. https://seer.cancer.gov/statfacts/html/all.html.

[14] Eastham J.A., Auffenberg G.B., Barocas D.A., Chou R., Crispino T., Davis J.W., Eggener S., Horwitz E.M., Kane C.J., Kirkby E. (2022). Clinically localized prostate cancer: AUA/ASTRO guideline, part I: Introduction, risk assessment, staging, and risk-based management. J. Urol..

[15] National Comprehensive Cancer Network (2026). NCCN Clinical Practice Guidelines in Oncology: Prostate Cancer, Version 5.2026.

[16] Merhe A., Hout M., Abou Heidar N., El-Asmar J.M., Jaafar R., Mailhac A., Tamim H., Nasr R. (2020). Is age an independent risk factor for perioperative mortality and morbidity after radical prostatectomy? Analysis of the American College of Surgeons National Surgical Quality Improvement Program database. Arab J. Urol..

[17] Freitas P.F.S., Blachman-Braun R., Soodana-Prakash N., Williams A.D., Ritch C.R., Punnen S., Gonzalgo M.L., Parekh D., Nahar B. (2024). Changing times: Trends in risk classification, tumor upstaging, and positive surgical margins after radical prostatectomy—Results from a contemporary National Cancer Database study. World J. Urol..

[18] Mukherjee K., Kamal K.M. (2019). Variation in prostate surgery costs and outcomes in the USA: Robot-assisted versus open radical prostatectomy. J. Comp. Eff. Res..

[19] Ploussard G., Grabia A., Beauval J.B., Barret E., Brureau L., Dariane C., Fiard G., Fromont G., Gauthé M., Mathieu R. (2021). A 5-year contemporary nationwide evolution of the radical prostatectomy landscape. Eur. Urol. Open Sci..

[20] Maynou L., Mehtsun W.T., Serra-Sastre V., Papanicolas I. (2021). Patterns of adoption of robotic radical prostatectomy in the United States and England. Health Serv. Res..

[21] Labban M., Dasgupta P., Song C., Becker R., Li Y., Kreaden U.S., Trinh Q.D. (2022). Cost-effectiveness of robotic-assisted radical prostatectomy for localized prostate cancer in the UK. JAMA Netw. Open..

[22] Kim D.K., Moon Y.J., Chung D.Y., Jung H.D., Jeon S.H., Kang S.H., Paick S., Lee J.Y. (2025). Comparison of robot-assisted, laparoscopic, and open radical prostatectomy outcomes: A systematic review and network meta-analysis from KSER update series. Medicina.

[23] Lee D., Choi S.K., Park J., Shim M., Kim A., Lee S., Song C., Ahn H. (2015). Comparative analysis of oncologic outcomes for open vs. robot-assisted radical prostatectomy in high-risk prostate cancer. Korean J. Urol..

[24] Kadono Y., Nohara T., Kawaguchi S., Iwamoto H., Yaegashi H., Shigehara K., Izumi K., Mizokami A. (2022). Impact of pelvic anatomical changes caused by radical prostatectomy. Cancers.

[25] Jung J., Jo H.W., Kwon H., Jeong N.Y. (2014). Clinical neuroanatomy and neurotransmitter-mediated regulation of penile erection. Int. Neurourol. J..

[26] Costello A.J., Brooks M., Cole O.J. (2004). Anatomical studies of the neurovascular bundle and cavernosal nerves. BJU Int..

[27] Yoshimura N., Chancellor M.B. (2003). Neurophysiology of lower urinary tract function and dysfunction. Rev. Urol..

[28] Kinter K.J., Newton B.W. (2023). Anatomy, Abdomen and Pelvis, Pudendal Nerve. StatPearls.

[29] Abrams P., Cardozo L., Wagg A., Wein A. (2017). Incontinence.

[30] Yu K., Bu F., Jian T., Liu Z., Hu R., Chen S., Lu J. (2024). Urinary incontinence rehabilitation after radical prostatectomy: A systematic review and network meta-analysis. Front. Oncol..

[31] Hodges P., Stafford R., Coughlin G.D., Kasza J., Ashton-Miller J., Cameron A.P., Connelly L., Hall L.M. (2019). Efficacy of a personalised pelvic floor muscle training programme on urinary incontinence after radical prostatectomy (MaTchUP): Protocol for a randomised controlled trial. BMJ Open..

[32] Moschovas M.C., Patel V. (2022). Neurovascular bundle preservation in robotic-assisted radical prostatectomy: How I do it after 15,000 cases. Int. Braz. J. Urol..

[33] Furrer M.A., Sathianathen N.J., Mulholland C.J., Papa N., Katsios A., Soliman C., Lawrentschuk N., Peters J.S., Zargar H., Costello A.J. (2025). Pelvic lymph node dissection in prostate cancer: Is it really necessary? A multicentric longitudinal study assessing oncological outcomes in patients with prostate cancer undergoing pelvic lymph node dissection vs. radical prostatectomy only. J. Urol..

[34] Dong B., Zhan H., Luan T., Wang J. (2024). The role and controversy of pelvic lymph node dissection in prostate cancer treatment: A focused review. World J. Surg. Oncol..

[35] Pisipati S., Ali A., Mandalapu R.S., Haines G.K., Singhal P., Reddy B.N., Leung R., Tewari A.K. (2014). Newer concepts in neural anatomy and neurovascular preservation in robotic radical prostatectomy. Indian J. Urol..

[36] Mata L.R.F.D., Azevedo C., Izidoro L.C.R., Ferreira D.F., Estevam F.E.B., Amaral F.M.A., Chianca T.C.M. (2021). Prevalence and severity levels of post-radical prostatectomy incontinence: Different assessment instruments. Rev. Bras. Enferm..

[37] Holm H.V., Fosså S.D., Hedlund H., Schultz A., Dahl A.A. (2014). How should continence and incontinence after radical prostatectomy be evaluated? A prospective study of patient ratings and changes with time. J. Urol..

[38] Emanu J.C., Avildsen I.K., Nelson C.J. (2016). Erectile dysfunction after radical prostatectomy: Prevalence, medical treatments, and psychosocial interventions. Curr. Opin. Support. Palliat. Care..

[39] Resnick M.J., Koyama T., Fan K.H., Albertsen P.C., Goodman M., Hamilton A.S., Hoffman R.M., Potosky A.L., Stanford J.L., Stroup A.M. (2013). Long-term functional outcomes after treatment for localized prostate cancer. N. Engl. J. Med..

[40] Moretti T.B.C., Magna L.A., Reis L.O. (2024). Erectile dysfunction criteria of 131,350 patients after open, laparoscopic, and robotic radical prostatectomy. Andrology.

[41] Haglind E., Carlsson S., Stranne J., Wallerstedt A., Wilderäng U., Thorsteinsdottir T., Lagerkvist M., Damber J.E., Bjartell A., Hugosson J. (2015). Urinary incontinence and erectile dysfunction after robotic versus open radical prostatectomy: A prospective, controlled, nonrandomised trial. Eur. Urol..

[42] Xiong T.Y., Liu Z.L., Wu H.Y., Fan Y.P., Niu Y.N. (2025). Association between maximal urethral length preservation and postoperative continence after robot-assisted radical prostatectomy: A meta-analysis and systematic review. Asian J. Androl..

[43] Tsui O.W.K., Shing K.C.H., Lam A.P.M., Ng S.L., Chun S., Tsang C.F., Lai T.C.T., Na R., Wong H.L., Ho B.S.H. (2025). Effects of nerve sparing on erectile dysfunction and urinary incontinence in robot-assisted radical prostatectomy. Hong Kong Med. J..

[44] Lee J., Kim H.Y., Goh H.J., Heo J.E., Almujalhem A., Alqahtani A.A., Chung D.Y., Chang K., Choi Y.D., Rha K.H. (2020). Retzius-sparing robot-assisted radical prostatectomy conveys early regain of continence over conventional robot-assisted radical prostatectomy: A propensity score matched analysis of 1,863 patients. J. Urol..

[45] Burnett A.L., Nehra A., Breau R.H., Culkin D.J., Faraday F.F., Hakim L.S., Heidelbaugh J., Khera M., McVary K.T., Miner M.M. (2018). Erectile dysfunction: AUA guideline. J. Urol..

[46] Salonia A., Bettocchi C., Boeri L., Capogrosso P., Carvalho J., Cilesiz N.C., Cocci A., Corona G., Dimitropoulos K., Gül M. (2021). European Association of Urology guidelines on sexual and reproductive health—2021 update: Male sexual dysfunction. Eur. Urol..

[47] De Angelis L., Marfella M.A., Siniscalchi M., Marino L., Nappo F., Giugliano F., De Lucia D., Giugliano D. (2001). Erectile and endothelial dysfunction in Type II diabetes: A possible link. Diabetologia.

[48] McMahon C.N., Smith C.J., Shabsigh R. (2006). Treating erectile dysfunction when PDE5 inhibitors fail. BMJ.

[49] Bonanni M., Rehak L., Massaro G., Benedetto D., Matteucci A., Russo G., Esperto F., Federici M., Mauriello A., Sangiorgi G.M. (2022). Autologous Immune Cell-Based Regenerative Therapies to Treat Vasculogenic Erectile Dysfunction: Is the Immuno-Centric Revolution Ready for the Prime Time?. Biomedicines.

[50] Ding X.G., Li S.W., Zheng X.M., Hu L.Q., Hu W.L., Luo Y. (2009). The effect of platelet-rich plasma on cavernous nerve regeneration in a rat model. Asian J. Androl..

[51] Wu C.C., Wu Y.N., Ho H.O., Chen K.C., Sheu M.T., Chiang H.S. (2012). Neuroprotective effect of platelet-rich plasma on erectile function in a rat model of cavernous nerve injury. J. Sex. Med..

[52] Epifanova M.V., Chalyi M.E., Krasnov A.O. (2017). Investigation of mechanisms of action of growth factors of autologous platelet-rich plasma used to treat erectile dysfunction. Urologiia.

[53] Shan H., Chen F., Zhang T., He S., Xu L., Wei A. (2015). Stem cell therapy for erectile dysfunction of cavernous nerve injury rats: A systematic review and meta-analysis. PLoS ONE.

[54] Sun D.Z., Abelson B., Babbar P., Damaser M.S. (2019). Harnessing the mesenchymal stem cell secretome for regenerative urology. Nat. Rev. Urol..

[55] Sun C., Lin H., Yu W., Li X., Chen Y., Qiu X., Wang R., Dai Y. (2012). Neurotrophic effect of bone marrow mesenchymal stem cells for erectile dysfunction in diabetic rats. Int. J. Androl..

[56] Levy J.A., Marchand M., Iorio L., Cassini W., Zahalsky M.P. (2016). Determining the feasibility of managing erectile dysfunction in humans with placental-derived stem cells. J. Am. Osteopath. Assoc..

[57] Bieri M., Said E., Antonini G., Dickerson D., Tuma J., Bartlett C.E., Patel A.N., Gershman A. (2020). Phase I and registry study of autologous bone marrow concentrate evaluated in PDE5 inhibitor refractory erectile dysfunction. J. Transl. Med..

[58] Poulios E., Mykoniatis I., Pyrgidis N., Kalyvianakis D., Hatzichristou D. (2023). Platelet-rich plasma for the treatment of erectile dysfunction: A systematic review of preclinical and clinical studies. Sex. Med. Rev..

[59] Haahr M.K., Harken Jensen C., Toyserkani N.M., Andersen D.C., Damkier P., Sørensen J.A., Sheikh S.P., Lund L. (2018). A 12-month follow-up after a single intracavernous injection of autologous adipose-derived regenerative cells in patients with erectile dysfunction following radical prostatectomy: An open-label phase I clinical trial. Urology.

[60] Protogerou V., Michalopoulos E., Mallis P., Gontika I., Dimou Z., Liakouras C., Stavropoulos-Giokas C., Kostakopoulos N., Chrisofos M., Deliveliotis C. (2019). Administration of adipose derived mesenchymal stem cells and platelet lysate in erectile dysfunction: A single center pilot study. Bioengineering.

[61] Korf-Klingebiel M., Kempf T., Sauer T., Brinkmann E., Fischer P., Meyer G.P., Ganser A., Drexler H., Wollert K.C. (2008). Bone marrow cells are a rich source of growth factors and cytokines: Implications for cell therapy trials after myocardial infarction. Eur. Heart J..

[62] Beer L., Mildner M., Gyöngyösi M., Ankersmit H.J. (2016). Peripheral blood mononuclear cell secretome for tissue repair. Apoptosis.

[63] Yiou R., Hamidou L., Birebent B., Bitari D., Le Corvoisier P., Contremoulins I., Rodriguez A.M., Augustin D., Roudot-Thoraval F., de la Taille A. (2017). Intracavernous injections of bone marrow mononucleated cells for postradical prostatectomy erectile dysfunction: Final results of the INSTIN clinical trial. Eur. Urol. Focus..

[64] Rennert R.C., Sorkin M., Januszyk M., Duscher D., Kosaraju R., Chung M.T., Lennon J., Radiya-Dixit A., Raghvendra S., Maan Z.N. (2014). Diabetes impairs the angiogenic potential of adipose-derived stem cells by selectively depleting cellular subpopulations. Stem Cell Res. Ther..

[65] Kornicka K., Houston J., Marycz K. (2018). Dysfunction of mesenchymal stem cells isolated from metabolic syndrome and Type 2 diabetic patients as result of oxidative stress and autophagy may limit their potential therapeutic use. Stem Cell Rev. Rep..

[66] Lotan Y., Cadeddu J.A., Gettman M.T. (2004). The new economics of radical prostatectomy: Cost comparison of open, laparoscopic and robot assisted techniques. J. Urol..

[67] Okhawere K.E., Shih I.F., Lee S.H., Li Y., Wong J.A., Badani K.K. (2021). Comparison of 1-year health care costs and use associated with open vs. robotic-assisted radical prostatectomy. JAMA Netw. Open..

[68] Sugihara T., Yasunaga H., Horiguchi H., Matsui H., Fujimura T., Nishimatsu H., Fukuhara H., Kume H., Changhong Y., Kattan M.W. (2014). Robot-assisted versus other types of radical prostatectomy: Population-based safety and cost comparison in Japan, 2012–2013. Cancer Sci..

